# Shenfu injection in sepsis-induced acute gastrointestinal injury: a narrative review of mechanisms and current evidence

**DOI:** 10.3389/fphar.2026.1811495

**Published:** 2026-05-01

**Authors:** Xiufeng Pang, Feng Zhu

**Affiliations:** 1 Department of Emergency Intensive Care Unit, Yangpu Hospital, School of Medicine, Tongji University, Shanghai, China; 2 Department of Critical Care Medicine, Shanghai East Hospital, School of Medicine, Tongji University, Shanghai, China

**Keywords:** acute gastrointestinal injury, intestinal barrier, quality control, sepsis, Shenfu injection

## Abstract

Shenfu Injection (SFI) is a standardized botanical drug derived from two Chinese medicinal herbs *Panax ginseng C.A.Mey*. and *Aconitum carmichaelii Debeaux*. SFI is historically used for “restoring yang and reversing collapse.” It is now widely applied in the treatment of septic shock. The gastrointestinal tract is considered the “engine” of sepsis, and its injury can lead to mucosal barrier disruption and secondary infection. The aim of this narrative review was to summarize the mechanistic basis, preclinical findings and available clinical evidence regarding the role of SFI in sepsis-induced acute gastrointestinal injury (AGI), while critically appraising the strengths and limitations of the current evidence base. A structured literature search of major English- and Chinese-language databases was performed. Preclinical and clinical studies were screened according to predefined inclusion criteria. Due to substantial heterogeneity in study design, models, interventions and outcomes, a quantitative meta-analysis was not performed. Research indicates SFI exerts effects through multiple targets: inhibiting the Toll-like receptor 4/Nuclear factor kappa B signaling (TLR4/NF-κB) pathway to reduce intestinal inflammation; upregulating tight junction (TJ) proteins such as zonula occludens-1 (ZO-1) and Occludin, reducing epithelial cell apoptosis, and modulating immune molecules to protect the physical and immune barriers of the intestinal mucosa; improving microcirculation and tissue perfusion; and potentially modulating gut microbiota composition. Although available clinical studies suggest potential benefits in selected physiological and surrogate endpoints, but most are constrained by small sample size, single-center design, and insufficient methodological reporting. Careful attention is also warranted to safety considerations, particularly aconite-related toxicity, standardization of preparation, and quality control, also warrant careful attention. Overall, SFI shows potential in stabilizing hemodynamics and maintaining the intestinal barrier, but current clinical evidence remains insufficient to support definitive efficacy claims. Validation through high-quality multicenter randomized controlled trials and further mechanistic studies are needed.

## 1 Introduction

Sepsis remains a major cause of morbidity and mortality among critically ill patients. In the United States and globally, it exacts a staggering economic toll and drives alarmingly high mortality rates (Liu et al., 2014; Torio and Moore, 2016; Rudd et al., 2020). Among the organ dysfunctions triggered by sepsis, the gastrointestinal tract is often the earliest affected organ and is therefore considered central to organ dysfunction (Königsrainer et al., 2011). Therefore, timely restoration of gastrointestinal function is crucial for preventing further deterioration in sepsis patients. However, effective specific treatments for sepsis-induced AGI remain scarce, with clinical management primarily relying on supportive and symptomatic therapies that yield limited efficacy. In recent years, SFI has garnered significant attention for its potential role in improving physiological functions in sepsis patients. SFI, a standardized pharmaceutical formulation derived from *Panax ginseng C.A. Mey*. and *Aconitum carmichaelii Debeaux*, which are recognized medicinal materials in the Chinese Pharmacopoeia (Standard Number: WS3-B-3427-98). This pharmacopoeia basis provides an important foundation for identity, quality control, and regulatory standardization of the formulation. As a classic botanical drug, it exhibits significant effects in warming “yang,” boosting “qi,” promoting blood circulation, and resolving stasis. Clinically, it is frequently used as an adjunctive therapy for critical conditions including sepsis, shock, and cardiovascular diseases (Xu et al., 2020). Recent studies indicate that SFI can alleviate sepsis-induced intestinal barrier dysfunction by modulating inflammatory responses and improving microcirculation. Specifically, SFI effectively reduces levels of inflammatory mediators such as tumor necrosis factor-alpha (TNF-α) and interleukin-6 (IL-6), thereby mitigating intestinal inflammation (Li et al., 2025). Furthermore, SFI may enhance intestinal function by modulating the composition of the gut microbiota (Yang et al., 2025). Therefore, thoroughly investigating the role and mechanisms of SFI in sepsis-induced AGI holds significant clinical importance and provides novel insights and directions for future therapeutic strategies.

In this review, we summarize the pathophysiological basis of sepsis-induced AGI and discuss the potential mechanisms by which SFI may confer intestinal protection. We further differentiate preclinical from clinical evidence, critically appraise the quality and limitations of the currently available literature, and highlight key safety issues and future research priorities.

## 2 Methods of literature identification and study selection

This work was conducted as a narrative review with a structured literature search, rather than a formal systematic review. To improve transparency and reproducibility, we searched PubMed, Web of Science, Embase, CNKI, Wanfang Data, and VIP databases for studies published up to 1 April 2026. The search terms included combinations of (“bioactive drug Shenfu Injection” OR “Shen-Fu Injection” OR “Shenfu” OR “Shen-Fu” OR “参附注射液”) AND (“sepsis” OR “septic shock”) AND (“acute gastrointestinal injury” OR “AGI” OR “gastrointestinal dysfunction” OR “intestinal injury” OR “gut barrier” OR “intestinal permeability” OR “gut microbiota”). Synonyms and alternative spellings were also considered during the search process. Eligible publications included preclinical and clinical studies addressing SFI in the context of sepsis, septic shock, gastrointestinal dysfunction, intestinal barrier injury, or related mechanisms relevant to AGI. Reviews, conference abstracts without sufficient data, duplicate publications, and studies not directly related to SFI or sepsis induced AGI were excluded from the core evidence synthesis. Mechanistic studies on sepsis-related intestinal injury that did not directly investigate SFI were used selectively to provide pathophysiological context and were identified as indirect evidence where applicable. Titles and abstracts were screened first, followed by full-text review for potentially relevant articles. Data extracted included study design, model or patient population, intervention details, comparator, major outcomes, and key limitations. A formal meta-analysis was not performed because of substantial heterogeneity across the included studies in terms of experimental models, patient populations, intervention protocols, outcome measures, and reporting quality. Likewise, although this was not designed as a formal systematic review, we critically appraised common sources of bias in the included literature, including sample size limitations, incomplete methodological reporting, unclear randomization/blinding procedures, and selective outcome reporting.

## 3 Sepsis and AGI

**FIGURE 1:**
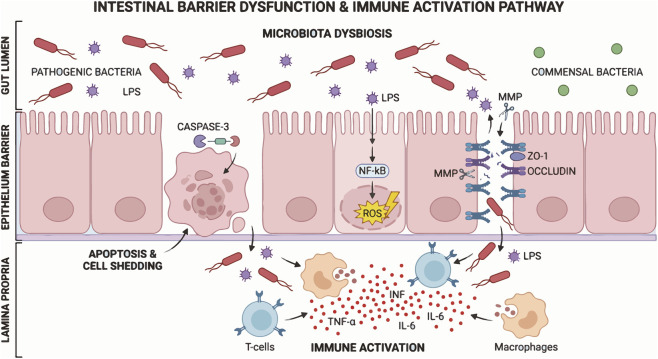
Mechanisms of sepsis-induced intestinal barrier dysfunction. In the gut lumen, microbiota dysbiosis (marked by excessive pathogenic bacteria and LPS leakage) disrupts the intestinal epithelial barrier structure. Within the epithelium, caspase - 3 induces apoptosis and cell shedding, while MMP - mediated degradation of TJ proteins (ZO - 1, Occludin) and NF -κB/ROS activation weaken barrier function further. Subsequently, in the lamina propria, immune cells such as T cells and macrophages are activated to secrete pro - inflammatory cytokines (TNF - α, IL - 6, INF), driving systemic immune responses.

Sepsis affects the gastrointestinal tract in multiple ways. It primarily promotes intestinal inflammation and impairs intestinal barrier function. It also reduces intestinal blood flow and triggers dysbiosis of the gut microbiota. This can lead to digestive dysfunction, and potentially cause severe complications such as intestinal necrosis (Figure 1). Sepsis severely compromises intestinal barrier function and markedly increases intestinal permeability. This promotes bacterial translocation and exacerbates systemic inflammatory responses, creating a vicious cycle that drives progressive deterioration in the patient’s condition and becomes a critical factor affecting prognosis.

### 3.1 Mechanisms of sepsis-induced intestinal barrier dysfunction

#### 3.1.1 Apoptosis and shedding of intestinal epithelial cells

Normal renewal and shedding of intestinal epithelial cells maintain intestinal barrier integrity. However, under pathological conditions such as sepsis and endotoxemia, apoptosis of intestinal epithelial cells significantly increases, leading to disruption of the intestinal barrier. Lai et al. (2013) observed that LPS induces accelerated migration and shedding of mouse intestinal epithelial cells, forming intercellular gaps that markedly increase intestinal permeability, with about 40% of leakage occurring through these gaps, suggesting that cell shedding is a key mechanism in intestinal barrier disruption. Additionally, the fungal metabolite gliotoxin (GT) similarly compromises intestinal barrier function by inducing IEC apoptosis. Upperman et al. (2003) observed in IEC-6 cells that GT significantly activated caspase-3 and promoted apoptosis in IEC-6 cells. This process was effectively blocked by pan-caspase inhibitors, which highlights the critical role of the apoptotic pathway in intestinal epithelial cell dysfunction. In addition, inflammasome signaling can exacerbate epithelial injury. Caspase-1 activation promotes the production of inflammatory cytokines such as IL-1β and IL-18, which contribute to epithelial cell extrusion, breaches in the intestinal barrier, and increased mucosal inflammation. Epithelial cell extrusion directly causes intestinal breaches, thereby linking cell loss to barrier dysfunction (Liu et al., 2013).

#### 3.1.2 Disruption of TJ proteins

The intestinal barrier which is made up of intestinal epithelial cells and their intercellular junctions; it is a complex structure. Serving as a physical barrier between the external and internal environment of the body, it plays a crucial role in maintaining homeostasis. The intestinal epithelial barrier is supported by multiple junctional complexes, including TJs, adherens junctions (AJs), desmosomes, and gap junctions (GJs), which together regulate epithelial cohesion, polarity, intercellular communication, and paracellular permeability (Alizadeh et al., 2022). Disruption to these junctional structures increases epithelial permeability, facilitating bacterial translocation and amplifying systemic inflammation. Among these, the TJ complex contains key proteins such as Occludin and ZO-1. ZO-1 dynamically regulates paracellular permeability by sealing intercellular gaps and controlling the selective transport of substances in the lumen, thereby ensuring normal intestinal function and barrier integrity (Xie et al., 2020). In a mouse study, results showed that intestinal permeability significantly increased after cecal ligation and puncture (CLP) sepsis modeling. After 12 h, Western blotting, PCR, and immunohistochemistry detected TJ proteins (claudin-1, 2, 3, 4, 5, 7, 8, 13, 15; JAM-A, Occludin, ZO-1). Sepsis increased Claudin-2 and JAM-A expression but reduced Claudin-5 and Occludin levels (Yoseph et al., 2016). Zhang et al. (2020) reported that MitoQ pretreatment restored Lipopolysaccharide (LPS) induced ZO-1 and Occludin expression, mitigating intestinal barrier damage. Furthermore, cyclooxygenase-2 (COX-2) and its product prostaglandin-2 are crucial for epithelial cell and barrier function, COX-2 deficiency increases intestinal permeability by reducing TJ protein, leading to exacerbated bacterial translocation and higher mortality in COX-2 knockout mice following CLP surgery (Fredenburgh et al., 2011). With increased intestinal permeability, the risk of bacterial translocation becomes greater, posing a severe threat to the intestinal mucosal system (Shimizu et al., 2011).

#### 3.1.3 Oxidative stress and inflammatory signaling pathways

Oxidative stress is widely recognized as a major driver of intestinal barrier dysfunction, exerting a significant and undeniable influence. Zhang et al. (2020) founded that MitoQ significantly mitigated LPS-induced oxidative damage by activating the Nrf2 signaling pathway and its downstream antioxidant genes, such as HO-1, NQO-1, and GCLM, showcasing its potential in protecting intestinal health. Concurrently, inflammatory mediators like TNF-α, IL-1β, and IL-6 play pivotal roles in intestinal barrier injury. Song et al. (2015) discovered that Cathelicidin-BF effectively mitigates intestinal inflammation by suppressing TNF-α expression through inhibition of the NF-κB signaling pathway, further revealing its crucial role in regulating intestinal inflammation. Furthermore, Wu et al. (2009) reported that the appetite-stimulating hormone ghrelin alleviates intestinal barrier dysfunction by activating the vagus nerve and reducing high-mobility group box 1 (HMGB1) levels. Oxidative stress has been linked to inflammatory signaling in cases of sepsis-induced intestinal injury. Excessive reactive oxygen species can damage lipids, proteins and mitochondrial function, thereby activate inflammatory pathways and promote cytokine release. Jia et al. found that inhibiting HMGB1 blocks ferroptosis and oxidative stress, thereby ameliorating sepsis-induced acute lung injury and significantly reducing the levels of IL-1β and IL-18 in the LPS-treated MLE-12 cell supernatant (Jia et al., 2024). Notably, LPS also induces matrix metalloproteinase-7 (MMP7) expression and Paneth cell degranulation, leading to significantly increased intestinal permeability. MMP7 acts as an inflammatory amplifier, with deficient mice exhibit weaker responses to intestinal inflammatory reactions. MMP7 activates α-defensins, thereby stimulating IL-6 release from macrophages and ileal epithelium, further exacerbating local intestinal inflammation and injury (Vandenbroucke et al., 2014). Microcirculatory disturbances are a hallmark of sepsis-associated gastrointestinal injury. Clinical imaging shows that sepsis alters intestinal mucosal microcirculation, which impairs oxygen delivery and causes epithelial hypoxia. This exacerbates barrier dysfunction (Schmidt et al., 2013).

#### 3.1.4 Microbiome dysbiosis and immune activation

Like the skin, the digestive and respiratory tracts possess large surface areas and perform complex physiological functions. These functions require a uniquely regulated immune system: the mucosal immune system (MIS) (Mowat and Agace, 2014). Within the gut, the MIS can distinguish between routine nutrient flow, self-antigens, diverse symbiotic microbial environments, and invading pathogenic microorganisms (Ahluwalia et al., 2017). An imbalance in the symbiotic gut microbiota-host relationship can threaten health. This involves abnormal activation of intestinal immune cells (e.g., CD4^+^, CD8^+^ T cells, Th17 cells, neutrophils, dendritic cells, macrophages) and depletion of regulatory T cells, promoting intestinal inflammation and barrier dysfunction. This allows bacteria and endotoxins to enter the bloodstream, triggering “viral sepsis” and multisystem injury. Major bacterial populations in the gut include commensal bacteria providing primary host energy (Wang et al., 2024). The commensal bacterium Ackermannia effectively mitigates inflammation by enhancing the stability of the intestinal epithelial barrier and suppressing the release of inflammatory cytokines (Lyv et al., 2024). However, when the intestinal barrier is compromised, bacterial translocation, endotoxin release, and dysbiosis can exacerbate intestinal inflammation. This excessive inflammatory response further impairs barrier function, creating a vicious cycle.

### 3.2 Sepsis and digestive function

Sepsis not only impacts the immune system but also causes complex gastrointestinal motility disorders, worsening prognosis with multiple organ dysfunction syndrome (MODS) and mortality. A clinical study on septic patients with AGI found that Acute Physiology and Chronic Health Evaluation II (APACHE II) and SOFA scores positively correlate with AGI severity, indicating increased mortality risk (Hai et al., 2024). Patients with AGI Grade IV exhibited significantly higher APACHE II and SOFA scores than those with lower AGI grades, with a 28-day mortality rate of 44.4%, compared to only 5.0% for AGI Grade I (Li et al., 2016). Further studies suggest that AGI grading correlates positively with ARDS severity. Multivariate analysis indicates that SOFA scores, the oxygenation index (PaO_2_/FiO_2_), the worst AGI value over 7 days and the SOFA score are independent risk factors for mortality within 28 days in AGI patients. For example, an increase of 1 point in the SOFA score raises the risk of death by 1.384-fold (Xu et al., 2024).

Another clinical study analyzing the impact of AGI grading on the prognosis of critically ill patients found that higher AGI grades (III-IV) were associated with higher D-dimer levels and elevated inflammatory markers, Both AGI III/IV and FI were associated with worse outcomes (Drakos et al., 2022). Another clinical study found sepsis patients had higher Gastrointestinal Dysfunction Score (GIDS) ≥2, feeding intolerance (FI) incidence rates and duration, elevated serum D-lactic acid, intestinal fatty acid-binding protein (I-FABP), IL-6, WBC, CRP, PCT, and longer ICU stays and ventilator days (Sun et al., 2024). FI is one manifestation of sepsis-induced gastrointestinal motility disorders, which include delayed gastric emptying, inhibited colonic contractions and prolonged intestinal transit times. Severe complications like intestinal obstruction may also occur (Ukleja, 2010). Studies indicate that gastrointestinal motility disorders affect 50%–80% of critically ill patients and can lead to multiple organ dysfunction (Govil and Pal, 2020; Adike and Quigley, 2014). Another study showed sepsis alone can delay gastrointestinal transit. Sepsis immediately after hemorrhagic shock worsened motility disorders, while later sepsis reduced severity, suggesting hemorrhagic shock’s protective pretreatment effect highlights the importance of injury sequence (Overhaus et al., 2009). Similarly, studies show septic patients have higher rates of FI and elevated serum diamine oxidase (DAO), D-lactic acid, and I-FABP levels. Moderate hypocaloric feeding may help these conditions but not necessarily reduce mortality (Sun et al., 2021).

Sepsis-induced AGI is not only a complex clinical issue with symptoms like abdominal pain, diarrhea, vomiting, and indigestion, potentially progressing to shock and multiple organ failure (MOF). As its presentation may resemble other gastrointestinal diseases, clinicians must be highly vigilant for timely diagnosis and effective treatment. Traditional Chinese Medicine (TCM) theory emphasizes clearing heat, detoxifying, restoring intestinal motility, promoting blood circulation, and enhancing immunity in sepsis treatment (Zhao et al., 2017). Multiple TCM interventions show efficacy in treating sepsis-induced AGI, reducing incidence and mortality through decreased inflammation and improved barrier function (Wang et al., 2017; Xing et al., 2019). A meta-analysis found TCM added to conventional care lowered mortality, ICU stay, and inflammatory markers (Liang et al., 2015). Electroacupuncture at specific points reduced mortality and ventilation time, especially in severe AGI (Yu et al., 2025). A systematic review highlights TCM’s benefits for motility, inflammation, and barrier repair, recommending individualized use and further research (Sang et al., 2025).

## 4 Organ protection effects of SFI in sepsis

### 4.1 Introduction to SFI

Among these TCM modalities, SFI is a modern, standardized preparation derived from two types of processed Chinese herbal medicine: red ginseng and radix aconiti lateralis praeparata. Red ginseng is processed from the dried root or other medicinal parts of *Panax ginseng C.A. Meyer*, a plant in the Araliaceae family which was first described by Carl Friedrich von Ledebour. Radix aconiti lateralis praeparata is processed from the root of *Aconitum carmichaelii Debeaux*, a plant in the Ranunculaceae family which was first described by Debeaux. SFI is essentially a TCM injectable preparation. Regarding the red ginseng metabolites, common bioactive metabolites include ginsenosides, such as Rg1, Re, Rb1, Rc, Rd, etc. For the radix aconiti lateralis praeparata metabolites, common bioactive or toxicity-related metabolites include: aconitine alkaloids, diester-type diterpene alkaloids, monoester-type diterpene alkaloids, as well as hydrolysis products of aconitine derivatives (He et al., 2014). These metabolites are rich in bioactive molecules that exert therapeutic effects through diverse mechanisms, demonstrating remarkable clinical efficacy particularly in treating sepsis and related acute gastrointestinal injuries (Xu et al., 2020). As a parenteral preparation, SFI is administered mainly by intravenous infusion, which enables rapid systemic delivery. Based on official drug instructions and related medical information sources, it is primarily indicated for conditions corresponding to “qi” deficiency and “yang” collapse in traditional Chinese medicine theory, and it has been used in the adjunctive management of shock, severe cardiovascular disorders, and critical illness. Research in network pharmacology research has also shown the complex interactions between SFI’s bioactive metabolites and multiple targets and signaling pathways. This provides support for its potential use in treating sepsis and opens up new avenues for future research and clinical applications (Yuan et al., 2022).

### 4.2 Role of SFI in circulation

**FIGURE 2:**
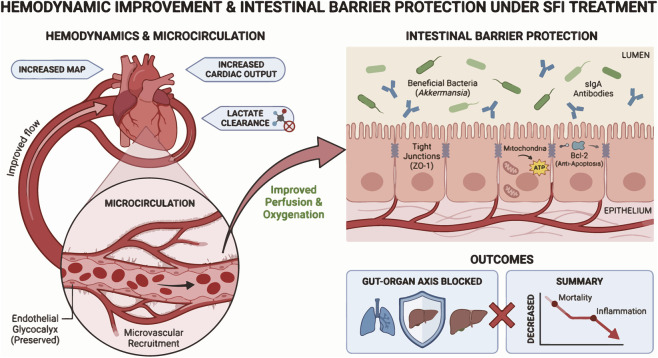
Effects of SFI on macroscopic and microscopic blood flow. SFI repairs the damaged intestinal epithelial barrier by improving systemic hemodynamic status through elevating mean arterial pressure (MAP), increasing cardiac output, and promoting lactate clearance. It optimizes intestinal microcirculatory perfusion efficiency, upregulates TJ protein ZO-1 expression, enhances mitochondrial ATP production, and strengthens Bcl-2-mediated anti-apoptotic effects to maintain intestinal epithelial integrity. Simultaneously improving gut microbiota balance and strengthening the sIgA immune barrier function. This blocks the gut-organ axis injury pathway, thereby achieving therapeutic goals of reducing mortality and suppressing systemic inflammation.

Sepsis is characterized by “hemodynamic dysregulation,” whereby restoring macrocirculation does not necessarily guarantee microcirculatory reperfusion, leading to tissue hypoxia and organ dysfunction. SFI exhibits dual efficacy: it stabilizes systemic hemodynamics while recruiting microvessels and protecting endothelial integrity (Figure 2).

For macro circulation, standard resuscitation guidelines for septic shock prioritize restoring mean arterial pressure (MAP) and cardiac output (CO). Multiple meta-analyses and randomized controlled trials (RCTs) confirm that adding SFI to standard combined therapy significantly improves macro circulatory parameters (Table 1). Both Mou et al.’s (2015) systematic review of 12 RCTs and Liao et al.’s (2024) large-scale meta-analysis of 56 RCTs confirmed SFI with conventional therapy significantly elevated MAP and restored heart rate (HR) better than conventional therapy alone. Both meta-analyses suggested that SFI reduced serum lactate levels, indicating improved tissue metabolic function. Another study indicates that SFI slows HR and increases MAP in septic rats within 3–12 h, returning to levels indistinguishable from controls after 12 h, suggesting short-term hemodynamics improvement, differing from the previous two studies (Pan et al., 2019). In a multicenter RCT with 188 patients, Liu et al. (2024) found that the SFI group had lower serum lactate levels than the control group at 6, 12 and 24 h, indicating effective reversal of tissue hypoxia. The trial also identified SFI’s key advantage as its “vasopressor-sparing” effect. Fan et al. (2019) also showed that SFI significantly improved cardiac index (CI) and global end-diastolic volume index (GEDVI).

**TABLE 1:** Summary of evidence on hemodynamic effects of SFI studies.

Study	Mou et al. (2015)	Liao et al. (2024)	Pan et al. (2019)	Liu et al. (2024)	Fan et al. (2019)	Wang et al. (2022)	Wu et al. (2016)
Study type	Meta-analysis and Systematic Review	Meta-analysis	Animal Study (Rat)	Multi-center RCT	Clinical Trial	RCT	Animal Study (Porcine)
Model	Clinical (12 RCTs, 904 patients)	Clinical (56 RCTs, 4,279 patients)	*In vivo* (SD rat endotoxemia model)	Clinical (188 septic patients)	Clinical (65 septic shock patients)	Clinical (40 septic shock patients)	*In vivo* (female Landrace pig VF-CPR model)
Dose range tested	Clinical: 50 mL SFI q12 h	Varies by administration route	SFI: 20 mL/kg IV	SFI 60 mL/d iv for 5–7 days	SFI 100 mL iv q12 h	SFI 100 mL iv for 5 days	1 mL/kg iv (1 h pre-VF)
Controls used	Conventional therapy	Routine Western therapy	Untreated control	Standard bundle therapy	Conventional therapy	5% glucose injection	Saline
Extract type	Source: *Aconitum carmichaeli*, *Panax ginseng*	Source: *Panax ginsengC.A.Mey*. + *Aconitum carmichaeli*	Source: *Panax ginseng* + *Aconitum carmichaeli*	Source: *Radix ginseng* + *Radix Aconiti Lateralis Preparata*	Source: *Radix Ginseng Rubra* + *Radix Aconiti Lateralis Preparata*	Source: *Panax ginseng* + *Aconitum carmichaeli*	Source: *Panax ginseng* + *Aconitum carmichaeli*
Basic pharmacological data	Reduced 28-day mortality (HR = 0.749), shortened vasopressor/MV/ICU time, improved MAP/HR/lactate clearance	Reduced 24 h/72 h/7 d SOFA, 14 d/28 d mortality, increased 6 h/24 h MAP, lactate	Improved intestinal epithelial mitochondrial function, reduced inflammation	Shortened vasopressor use, reduced 12 h/24 h lactate	Increased CI, GEDI, MAP, decreased HR at 12/24 h; No change in ELWI	Reduced APACHE/SOFA scores, shortened EICU stay, 28-day mortality 15% vs. 25%	Fewer defibrillations, shorter CPR duration, higher CPP during CPR/ROSC
Taxonomic validity	Confirmed: DNA barcode + pharmacopoeia standards; Standardization: GMP-certified (Huaren Sanjiu), patented process (ZL96117458.7), CFDA-approved (WS3-B-3427-98–2013)	Confirmed: *Panax ginseng* + *Aconitum carmichaelii*; Standardization: Not detailed	Confirmed: *Panax ginseng* + *Aconitum*.Standardization: China Ministry of Health standards	Confirmed: Traditional formula (Shenfu Decoction, *Jisheng Fang*) Standardization: Not detailed	Confirmed: *Radix Ginseng Rubra* + *Radix Aconiti Lateralis Preparata*Standardization: Manufacturer (Ya’an Sanjiu), batch number	Confirmed: Traditional formula (Shenfu Decoction); Standardization: State FDA approval (Z20043117)	Confirmed: *Panax ginseng* + *Aconitum carmichaeli*; Standardization: 4-in-1 QC (fingerprint, active ingredients, toxins, limits)
Model relevance	High	High	Moderate	High	High	High	Moderate

Although macro circulatory stability is crucial, microcirculatory dysfunction remains the primary driver of sepsis-induced organ injury. Studies on SFI’s microcirculatory effects also yielded positive outcomes. For instance, Wang et al. (2022) suggested SFI’s impact on microcirculation in septic shock patients using side-stream dark-field (SDF) imaging. Confirming it significantly increased total vascular density (TVD), perfusion vascular density (PVD), and the microvascular flow index (MFI). This effect may stem from endothelial protection, as SFI reduced plasma biomarkers like angiopoietin-2 (Ang-2), syndecan-1 (Syn-1), and endothelin-1 (ET-1). By inhibiting endothelial glycocalyx shedding and reducing vasoconstrictive endothelin, SFI helps maintain capillary patency and reduce vascular permeability. Wu et al. (2016) confirmed in a porcine shock model that SFI enhances microvascular blood flow in both mesenteric macro vessels and micro vessels, which is relevant to preventing AGI. As sublingual microcirculation is an indirect measure of intestinal function, further confirmation is required to establish the relevance of these findings to gastrointestinal outcomes. While the mesenteric circulation is anatomically linked to the GI tract, it does not fully represent the complex microvascular network of the gastric and intestinal mucosa, which is a critical site for nutrient absorption, barrier integrity and immune modulation.

However, some studies suggest that there are discrepancies between systemic and intestinal microcirculation, whereby improvements in systemic microcirculation do not enhance intestinal microcirculation. A study on rats showed that, 50 min after an intravenous injection of 1 × 10^9^ live *Escherichia coli*, cardiac output increased 20%, while systemic vascular resistance decreased 14%. Conversely, blood flow to the small intestine’s microvasculature decreased 27% within 1 h, plummeting a further 56% over the subsequent 2 h. This indicates progressive, significant arteriolar constriction (25%–50%) throughout the intestinal microcirculation. These data reveal that arteriolar constriction during high-cardiac-output sepsis may cause intestinal hypoperfusion, potentially triggering mucosal injury and barrier impairment, with profound systemic effects (Whitworth et al., 1989). Another study indicates that there is no significant correlation between systemic perfusion and intestinal circulation. Researchers analyzed systemic hemodynamics, sublingual microcirculation, and intestinal circulation in sepsis patients. Results showed sublingual microcirculation improvements were tied to cardiac output, but revealed a dissociation between sublingual and intestinal microcirculation, offering new insights into the complexity of microcirculatory regulation (Edul et al., 2014). Targeted studies using AGI-focused SDF imaging or intravital microscopy of intestinal villi are needed to quantify SFI’s effects on AGI microvascular parameters.

### 4.3 SFI and intestinal barrier function

**FIGURE 3:**
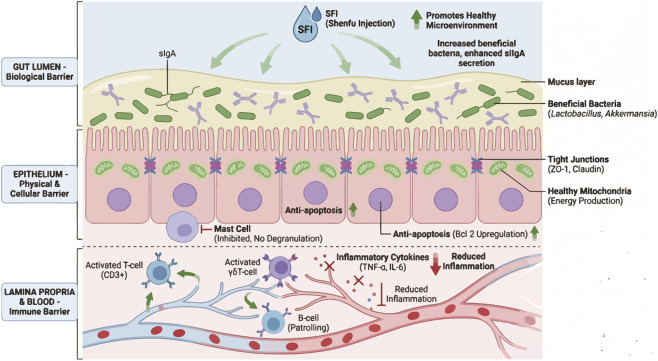
Effects and mechanisms of SFI on the intestinal barrier. Shenfu Injection (SFI) enhances gut health by promoting beneficial bacteria (Lactobacillus, Akkermansia) and sIgA secretion, fortifying the mucosal barrier; it strengthens epithelial integrity via TJ proteins (ZO - 1/Cludin), mitochondrial energy production, and anti - apoptotic factors (Bcl - 2), while inhibiting mast cell degranulation; systemically, SFI modulates immune responses (activating T/B cells, reducing TNF - α/IL - 6) to protect organs like lungs (via lung - gut axis) and liver (via reduced bacterial translocation).

Evidence constitutes the main body of direct support for the use of SFI in sepsis-induced AGI (Figure 3). However, the overall evidentiary strength remains limited. Many studies used small animal cohorts, incompletely reported randomization or blinding, and relied on histologic or biochemical surrogate endpoints rather than clinically translatable outcomes. In several reports, the mechanistic conclusions were largely based on associative changes in protein expression without genetic or pharmacologic pathway validation. Therefore, these studies support biological plausibility, but do not yet establish robust causal mechanisms (Table 2). Early observational studies on SFI’s protective effects on the intestinal mucosal barrier in septic rats revealed that SFI not only effectively ameliorates intestinal inflammatory responses and apoptosis in septic models but also significantly increases the expression levels of TJ proteins in intestinal epithelial cells (Wu et al., 2015). Xing et al. (2015) found that SFI suppresses inflammation by reducing TNF-α, improves tissue oxygen metabolism through antioxidant effects, and mitigates damage to TJs. It also directly repairs the intestinal barrier by upregulating Claudin-3 and ZO-1, restoring intercellular connections in epithelial cells.

**TABLE 2:** Studies of Shenfu-based interventions in sepsis-induced AGI.

Study	Study type	Model	Preparation	Dose range and route	Duration	Controls	Endpoints	Main findings	Evidence level/key limitations
Wu et al. (2015)	*In vivo* animal study	Rat sepsis model	Shenfu Injection (SFI)	5 mL/kg and 10 mL/kg, iv	8 h	NS	Intestinal mucosal barrier injury	SFI attenuated sepsis-induced intestinal mucosal barrier injury	Preclinical evidence; limited translational validity
Xing et al. (2015)	*In vivo* animal study	Rat sepsis model	Shenfu Injection (SFI)	5 mL/kg, 10 mL/kg, and 20 mL/kg, iv	12 h	NS	Intestinal epithelial injury	SFI mitigated intestinal epithelial damage	Preclinical evidence; mechanistic insight remains limited
Liu et al. (2021)	*In vivo* animal study	Rat sepsis model	Shenfu decoction (SFD)	3 mg/kg, gavage, twice daily	72 h	Distilled water	Intestinal permeability; barrier-associated proteins	SFD reduced intestinal permeability and modulated barrier-related protein expression	Causality not fully established
Zheng et al. (2022)	*In vivo* and *in vitro* study	AGI-aggravated ALI model; MLE-12 cells	Shenfu Injection (SFI)	Mice: 30 mL/kg, iv; cells: 10 μL/mL	7 days	NS	Gut–lung barrier dysfunction; claudin-4	SFI upregulated claudin-4 and alleviated lung injury	Multi-organ model; pathway validation remains insufficient
Jin et al. (2019)	*In vivo* animal study	Rat sepsis model	Shenfu Injection (SFI)	5 mL/kg and 20 mL/kg, iv	12/24 h	NS	Intestinal mucosal injury; survival	SFI prolonged survival and reduced intestinal mucosal injury	Preclinical evidence; mechanism broadly defined and clinically unverified
Li et al. (2025)	*In vivo* animal study	Mice sepsis model	Shenfu Injection (SFI)	200 μL, iv	16 h	NS	Intestinal inflammation, IL-1β, IL-6, SCr, BUN, gut microbiota	SFI reduced inflammation, improved microbiota dysbiosis	No direct functional AGI outcomes; causality of microbiota changes not proven
Wu et al. (2022)	*In vivo* animal study	Rat sepsis model	Shenfu Injection (SFI)	20 mg/kg	12 h	NS	Apoptosis, bacterial translocation, TJ	SFI reduced mast-cell activation and bacterial translocation and preserved barrier proteins	Good barrier evidence; short-term study, no survival outcome
Deng et al. (2025)	Clinical and animal study	Septic patients and mice sepsis model	Shenfu Injection (SFI)	Clinical: 50 mL/6 h for 5 days, ivgtt; mice: 10/20 mL/kg, ip	Clinical: 5 days; animal duration 48 h	NR	D-LAC, I-FABP, stem-cell renewal, TJ, microbiota	SFI improved epithelial repair; ATF4-related mechanism	Limited clinical causal evidence
Zhang et al. (2018)	Randomized clinical study	Sepsis patients	Shenfu Injection (SFI)	60 mL, ivgtt, once daily	2 weeks	Conventional treatment	I-FABP, soluble CD14 subtype, MDA, SOD	SFI improved intestinal barrier and oxidative stress; survival improved	Clinically relevant biomarker study; single-center, limited AGI outcomes

Abbreviations: SFI, Shenfu injection; SFD, Shen-Fu Decoction; AGI, acute gastrointestinal injury; ALI, acute lung injury; ATF4, activating transcription factor 4; BUN, blood urea nitrogen; D-LAC, D-lactate; I-FABP, intestinal fatty acid-binding protein; IL, interleukin; ip, intraperitoneal; iv, intravenous; ivgtt, intravenous drip; MDA, malondialdehyde; MLE-12, murine lung epithelial-12 cells; NR, not reported; NS, normal saline; SCr, serum creatinine; SOD, superoxide dismutase.

Quantitative data were not extracted here and should be verified from the full-text articles before formal use in a manuscript.

An animal study on oral administration of Shenfu Decoction showed it modulates proteins like ZO-1, Occludin, Claudin-1, and p-VASP, reducing mortality in CLP rats, preventing intestinal and hepatic injury, alleviating sepsis-induced permeability and inflammation, and improving intestinal permeability impairment (Liu et al., 2021). Zheng et al. (2022) showed SFI’s protective effect on intestinal epithelium indicates systemic action via the lung-gut axis, consistent with the TCM theory of “the lung and intestine are paired organs.” The composite model (hemorrhagic shock + LPS) used here better approximates clinical ALI’s multifactorial etiology than single-factor models. In the AGI + ALI composite model, SFI significantly alleviated lung and intestinal tissue damage and restored claudin-4 expression.

SFI also significantly enhances the innate immunity, upregulating sIgA levels and increasing CD3 and γδ T cells in a dose-dependent manner, improving intestinal mucosal damage and inflammatory, prolonging septic rat survival and offering evidence for clinical adjunctive treatment of sepsis (Jin et al., 2019). An animal study showed that SFI reduced intestinal injury, mast-cell activation, epithelial apoptosis, and bacterial translocation in CLP-induced septic rats. It also increased the expression of TJ proteins such as Occludin and ZO-1, suggesting preservation of barrier integrity. Notably, SFI performed better than ketotifen fumarate on several gut-related outcomes. The main limitations are the short observation period and the lack of long-term functional or survival endpoints (Wu et al., 2022).

SFI also exerts therapeutic effects against sepsis by reshaping gut microbiota composition, suppressing systemic inflammatory responses, and protecting the intestinal mucosal barrier. In the SFI-treated sepsis group, villus arrangement restored regularity, mucosal integrity markedly improved, and levels of renal injury markers (SCr, BUN) decreased. The promotion of pro-inflammatory factors resulted in a 10.3-fold decrease in IL-1β and a 3.8-fold decrease in IL-6, while enriching beneficial probiotic bacteria (e.g., *Akkermansia* and *Lactobacillus*). Microbiota-inflammation correlation analysis revealed significant negative correlations between Enterococcus abundance and IL-1β/IL-6, and positive correlations between *Ruminococcus* and *Bacteroides* with IL-6 (Li et al., 2025). However, this evidence is still preliminary and should be interpreted as hypothesis-generating.

Clinical studies also show the efficacy of SFI in improving intestinal barrier function during sepsis. But most available studies assessed surrogate indicators rather than direct and standardized AGI outcomes, which limits the certainty of clinical inference. A translational study suggests that SFI may directly protect the gut in sepsis by promoting intestinal epithelial regeneration. In septic mice, SFI improved mucosal injury, increased TJ proteins, reduced epithelial apoptosis, and decreased inflammatory cytokines, while also modulating gut microbiota composition. The authors proposed that these effects were mediated, at least partly, through ATF4-dependent intestinal stem cell self-renewal. However, the human evidence was mainly biomarker-based, and the causal role of ATF4 and microbiota changes still requires stronger validation (Deng et al., 2025). An investigation into SFI’s therapeutic effects and mechanisms for treating intestinal mucosal barrier dysfunction in sepsis suggested pathways, including protecting the intestinal barrier; reducing I-FABP levels, modulating immune responses by decreasing soluble CD14 subtype levels; reducing oxidative stress; lowering MDA levels; increasing SOD activity; improving tissue perfusion; improving the 28-day survival rate (Zhang et al., 2018). However, the study was single-center, the sample size was modest, and major clinical outcomes such as feeding tolerance, AGI grade evolution, and ICU length of stay were not fully reported.

A major limitation of the above evidence is that the benefits of SFI in sepsis-induced AGI are mainly supported by indirect evidence. Most studies assessed surrogate indicators such as I-FABP, lactate, oxidative stress markers, intestinal histology or tight-junction proteins rather than direct AGI outcomes such as AGI grade, tolerance of enteral feeding, gastrointestinal failure or AGI-related mortality. Furthermore, many studies were single-center, involved small samples, were short-term, and were methodologically heterogeneous. Therefore, while the existing data suggest a possible gut-protective effect of SFI, they are insufficient to confirm its definitive efficacy in treating sepsis-related AGI.

## 5 Impact of SFI on sepsis-induced AGI

In the early stages of sepsis, sepsis-induced AGI may already reveal its potential threat, clinically manifesting as a series of severe symptoms including malignant vomiting, diarrhea, abdominal distension, and abdominal pain. These may even be accompanied by gastrointestinal bleeding, organ dysfunction or failure, and in extreme cases, may lead to critical conditions such as intestinal obstruction and intestinal necrosis. AGI severity is typically categorized into four levels (AGI I-IV) (Reintam Blaser et al., 2021a) (Table 3). However, AGI grading is not merely a subjective assessment of patient condition; it specifically focuses on the concept of FI, which itself relies on local feeding practices and management and is relatively poorly defined. Consequently, the subjective judgment of observers plays a particularly important role in this process. At the same time, patients with more severe conditions are often assigned higher AGI grades compared to those exhibiting similar gastrointestinal symptoms but milder disease (Reintam Blaser et al., 2012). A novel five-tier Gastrointestinal Dysfunction Score (GIDS) was developed by Reintam Blaser et al. for these reasons, in a multicenter prospective study (Table 4). The study found that GIDS was strongly correlated with independent 28-day and 90-day outcomes. Compared to traditional SOFA total scores or models incorporating all SOFA scores, GIDS showed significantly enhanced predictive capability across all analyses (Reintam Blaser et al., 2021b). Beyond clinical manifestations, research indicates that biomarkers such as D-lactic acid, I-FABP, and citrulline can be utilized to assess AGI severity (Tyszko et al., 2023). Current research on SFI’s role in improving sepsis-induced acute gastrointestinal dysfunction primarily focuses on its indirect effects through enhanced intestinal barrier function. Extensive animal and clinical studies indicate that SFI significantly increases the expression of TJ markers in intestinal epithelial cells, thereby effectively mitigating the extent of intestinal epithelial injury.

**TABLE 3:** Grading criteria and clinical management of AGI.

AGI grade	Definition and clinical features	Clinical examples	Management principles
AGI I (Risk of GI dysfunction or failure)	Partial impairment of gastrointestinal function. Manifested as transient gastrointestinal symptoms with clear etiology	Early postoperative nausea/vomiting Absence of bowel sounds after surgery Reduced intestinal peristalsis in early shock	Condition usually improves spontaneously without specific intervention Early enteral nutrition recommended within 24–48 h Limit use of medications that impair gastrointestinal motility when possible
AGI II (GI dysfunction)	The gastrointestinal tract cannot adequately perform digestion and absorption to meet the body’s nutritional and fluid requirements. The patient’s overall condition has not changed due to GI issues	Gastroparesis with large gastric retention/reflux Low intestinal paralysis Diarrhea Grade I intra-abdominal hypertension (IAP 12–15 mmHg) Feeding intolerance (unable to achieve 20 kcal/kg/day EN within 72 h)	Therapeutic measures needed to prevent progression to GI failure (e.g., treat intra-abdominal hypertension, use prokinetic agents) Initiate or continue enteral nutrition For gastric retention/intolerance, consider small-volume feeding trials post-pyloric feeding may be considered for gastroparetic patients
AGI III (GI failure)	Loss of GI function. Despite interventions, recovery is not achieved and the patient’s overall condition does not improve	Persistent feeding intolerance after treatment IAH progressing to grade II (IAP 15–20 mmHg) Decreased abdominal perfusion pressure (APP) (<60 mmHg) Associated with persistent or worsening MODS	Monitor and treat IAH to prevent deterioration Rule out occult abdominal pathology Discontinue medications causing GI paralysis when possible Avoid early supplemental parenteral nutrition within 7 days of ICU admission Regular small-volume enteral nutrition trials
AGI IV (GI failure with severe impact on distant organs)	AGI progresses to a directly life-threatening state with worsening MODS and shock	Intestinal ischemia with necrosis Massive gastrointestinal bleeding causing hemorrhagic shock Ogilvie syndrome Abdominal compartment syndrome (ACS) requiring decompression	Surgical intervention or other emergency procedures required for life-saving (e.g., laparotomy, colonoscopic decompression) No proven conservative treatment options

**TABLE 4:** Severity grading of the gastrointestinal dysfunction score (GIDS).

Risk level	Description
0 - No Risk	Asymptomatic or presence of any one of the following symptoms under oral feeding: absence of bowel sounds, vomiting, GRV > 200 mL, gastrointestinal paralysis/motility disorders, abdominal distension, diarrhea (non - severe), no need for blood transfusion for gastrointestinal bleeding, IAP 12–20 mmHg
1 - Increased Risk	Presence of any two of the following symptoms: inability to feed orally, absence of bowel sounds, vomiting, GRV > 200 mL, gastrointestinal paralysis/motility disorders, abdominal distension, diarrhea (non - severe), no need for blood transfusion for gastrointestinal bleeding, IAP 12–20 mmHg
2 - Gastrointestinal Dysfunction	Presence of three or more symptoms in level 1, or presence of one to two of the following symptoms: severe diarrhea, gastrointestinal bleeding requiring blood transfusion, IAP > 20 mmHg, abdominal distension, severe diarrhea, IAP > 20 mmHg
3 - Gastrointestinal Failure	Presence of any three or more of the following symptoms: use of prokinetic agents for the gastrointestinal tract, gastrointestinal paralysis/motility disorders, abdominal distension, severe diarrhea, IAP > 20 mmHg
4 - Life - threatening	Presence of any one of the following conditions: gastrointestinal bleeding leading to hemorrhagic shock, mesenteric ischemia, abdominal compartment syndrome

Abbreviations: GRV, gastric residual volume; IAP, intra-abdominal pressure.

According to TCM theory, red ginseng serves as the “Monarch” (Jun) herb in SFI, functioning primarily to “vigorously reinforce Primordial Qi” (Yuan Qi). Biologically, this “Qi-tonifying” effect parallels the enhancement of cellular bioenergetics and systemic perfusion, providing the metabolic foundation for organ recovery. Concomitantly, radix aconiti lateralis praeparata acts as the “Minister” (Chen) herb, responsible for “restoring Yang to rescue collapse.” In TCM pathology, this action is analogous to reigniting the “Fire of the Vital Gate,” which provides the thermal energy essential for the Spleen and Stomach’s functions of transformation and transportation. Biologically, this “Yang-tonifying” effect parallels the correcting visceral hypoperfusion (e.g., mesenteric microcirculation) and improving mitochondrial exhaustion. The synergistic pairing of red ginseng and radix aconiti lateralis praeparata embodies the principle of “mutual engendering of Qi and Yang”. Biologically, this effect of “restoring vital energy and reviving yang energy” corresponds to the metabolic basis for enhancing organ function and creating an optimal internal milieu for restoring gastrointestinal motility and mucosal integrity (Zhang et al., 2016).

However, there is limited clinical evidence directly evaluating SFI in sepsis-induced AGI. The available studies are few in number, are often single-center and are heterogeneous in terms of patients, intervention protocols and outcome measures. Therefore, any clinical interpretation should be considered preliminary (Table 5). For instance, a recent randomized trial conducted in a single center reported that the early adjunctive administration of SFI was associated with lower serum pepsinogen I/II and gastrin-17 levels, as well as shorter durations of mechanical ventilation and ICU stay (Zhang et al., 2024). However, the study was limited by its single-center design, modest sample size and use of surrogate biomarkers rather than validated AGI severity scores or long-term gastrointestinal outcomes. Another clinical study showed that SFI was associated with an improvement in gastrointestinal dysfunction in patients with septic shock. Compared with conventional treatment, SFI reduced lactate and Lac/SCr levels, suggesting improved tissue perfusion and potentially enhanced gut function. It also appeared to relieve gastrointestinal symptoms and suppress inflammatory responses. However, the study was not a high-quality randomized blinded trial, and no clear survival benefit was demonstrated (Yuan et al., 2025). Therefore, clinical research still faces the following challenges. Limited level of clinical evidence: existing studies are predominantly single-center randomized controlled trials (RCTs) with small sample sizes, lacking multi-center large-scale validation. Insufficient mechanistic research: limited understanding of the specific molecular mechanisms by which SFI modulates intestinal microcirculation and the immune microenvironment. Optimization of timing and dosage: animal studies suggest earlier intervention yields better outcomes, but the optimal clinical administration regimen remains unclear. Lack of long-term prognosis assessment: existing studies predominantly focus on short-term indicators (e.g., ICU length of stay) and lack follow-up on long-term complications (e.g., enteric infections, organ dysfunction). Future research should prioritize: high-quality clinical studies: conduct multicenter RCTs to clarify efficacy differences across AGI severity grades (e.g., AGI II-IV) and explore synergistic effects with other TCM (e.g., rhubarb) or western medicines (e.g., probiotics). Mechanism innovation: integrating gut microbiota metagenomics and metabolomics technologies to decipher the molecular networks through which SFI modulates AGI via the “gut-liver axis” or “brain-gut axis.”

**TABLE 5:** Summary of two clinical studies on SFI for sepsis-induced AGI.

Item	Zhang et al. (2024)	Yuan et al. (2025)
Study design	Single-center RCT	Clinical observational study with PSM
Population	Sepsis patients with AGI	Septic shock patients with AGI
Intervention dose/route	SFI 100 mL/12 h	SFI 100 mL/12 h
Comparator/controls	Routine care	Conventional treatment
Treatment duration	7 days	5 days
AGI-related outcomes	PGI/PGII/G17; AGI grade; mechanical ventilation duration; ICU stay	GI symptom improvement; IL-6; Lac; Lac/SCr; 28-day mortality
Main findings	SFI lowered PGI by day 3, lowered PGI/PGII/G17 by day 7, reduced AGI grade, and shortened ventilation and ICU stay	SFI improved GI symptoms and reduced IL-6, Lac, and Lac/SCr, but did not reduce 28-day mortality
Major limitations	Single-center; modest sample size; short follow-up; gastric biomarkers are not yet standard AGI markers	Observational design despite PSM; residual confounding cannot be excluded

Abbreviations: AGI, acute gastrointestinal injury; G17, gastrin-17; GI, gastrointestinal; ICU, intensive care unit; IL-6, interleukin-6; Lac, lactate; PGI, pepsinogen I; PGII, pepsinogen II; PSM, propensity score matching; RCT, randomized controlled trial; SCr, serum creatinine; SFI, Shenfu injection.

## 6 Quality control and safety considerations of SFI as a botanical injectable

As an injectable botanical preparation of TCM derived from *Panax ginseng C.A.Mey*. and *Aconitum carmichaelii Debeaux*, the safety of SFI, particularly with regard to the aconite alkaloids metabolites, is a key focus of clinical attention. Another critical issue that requires explicit attention is the fact that SFI is a complex injectable preparation derived from botanical drugs and animal-derived materials. This raises quality and safety concerns that go beyond those associated with conventional small-molecule drugs. Batch-to-batch consistency, sterility assurance, endotoxin control, microbiological contamination, manufacturing standardization and chemical fingerprinting are all highly relevant for such preparations. These aspects are particularly important for critically ill patients with sepsis, who may be more vulnerable to adverse effects due to immune dysregulation and organ dysfunction. Although SFI has been used clinically in China for years, the published literature reviewed here provides limited systematic information on manufacturing consistency, contamination risk control and pharmacovigilance, particularly in cases of sepsis-associated AGI. Future reviews and clinical studies should therefore incorporate a more rigorous assessment of quality attributes and safety monitoring. Based on existing research, its safety data primarily stems from preclinical toxicology studies, pharmacokinetic analyses, randomized controlled clinical trials, and meta-analyses. Yang et al. (2025) conducted a long-term toxicity study of SFI, employing intraperitoneal injection due to technical difficulties associated with long-term, high-frequency intravenous administration. The researchers established three dose groups: High-dose (HSF): 12 g of raw herb per kg per day (12 times the clinical dose), Medium-dose (MSF): 9 g of raw herb per kg per day (9 times the clinical dose), Low-dose (LSF): 6 g of raw herb per kg per day (6 times the clinical dose). The treatment was administered continuously for 30 days, followed by a 28-day observation period (recovery period). Toxic responses were monitored using a comprehensive and systematic evaluation system, which included the following: general condition, body weight and food intake, ophthalmology and urinalysis, haematology and coagulation function, blood biochemistry (including hepatic, renal and cardiac function, as well as glucose and lipid profiles), systemic anatomy and organ indices, and histopathological examination. The results demonstrated that SFI has a wide safety margin, with controllable and reversible toxicity and no significant target organ toxicity. The article states that Ya’an Sanjiu, the manufacturer, conducted chemical fingerprinting analysis on the main chemical metabolites of SFI in order to achieve indirect control of consistency between batches. It also emphasizes that the drug complies with national drug standards and has clearly defined metabolites with controllable content. However, it does not provide comparative data between different batches, nor does it include an analysis of sterility assurance, endotoxin control or microbiological contamination. In the randomized controlled trial conducted by Zhang et al. (2017), SFI was administered at a dosage of 100 mL per infusion twice daily (BID). This treatment was administered via continuous intravenous infusion for 14 consecutive days to patients who had achieved return of spontaneous circulation (ROSC) following in-hospital cardiac arrest. Monitoring cardiac and consciousness status, recording adverse reactions such as vomiting and palpitations, and detecting QT interval prolongation and arrhythmias were critical components of the safety assessment. The study results indicated that no serious adverse drug reactions were reported in association with the administration of SFI at this dosage and regimen. Qiu et al. (2025) conducted a clinical trial, the dosing regimen consisted of 50 mL (alkaloids 2–12 μg/mL) of SFI diluted in 100 mL of normal saline, administered intravenously twice daily. The infusion rate was maintained at 40–60 drops per minute, and the treatment continued for five consecutive days. The study provided detailed information about the drug, including its manufacturer, approval number and specifications. The trial strictly adhered to the ‘Technical Guidelines for Causality Assessment of Adverse Events in Clinical Drug Trials (2024 Version).’ Safety was rigorously monitored through continuous assessment of vital signs, cardiovascular parameters and laboratory indicators. Any serious adverse events had to be reported to the Ethics Committee within 24 h. The incidence of adverse events was very low, including skin rash (one case), pruritus (one case), and mild nausea (three cases). All events were transient and did not require the treatment to be discontinued. Nausea was managed by adjusting the infusion rate. Importantly, none of the events were deemed “definitely related” or “probably related” to SFI according to the standardized causality assessment criteria. This paper did not include specialized analyses of toxicity, sterility, endotoxin control or microbiological contamination. Such data is usually found in the drug’s registration dossier or in pharmacopoeia standards, and was not presented within the scope of this clinical study. Liao et al.’s (2024) cite a real-world study involving 30,106 patients in their meta-analysis, which reported an incidence of adverse reactions of 0.76%. Additionally, three of the included studies documented adverse reactions such as increased heart rate and gastrointestinal reactions. However, the analysis does not elaborate on drug quality stability, microbiological contamination control or sterility assurance, nor does it provide experimental data on endotoxin control.

## 7 Limitations and future directions

Although existing studies suggest that SFI may protect against sepsis-induced AGI, the overall evidence remains limited. Most available data are preclinical, and the reported benefits are derived mainly from animal models using heterogeneous experimental settings, including endotoxemia, cecal ligation and puncture. These models differ in their relevance to human sepsis-induced AGI, which limits direct translation of the findings.

In addition, many preclinical studies rely on histology, inflammatory markers, or protein-expression changes as surrogate endpoints, while clinically meaningful gastrointestinal outcomes are less frequently assessed. Mechanistic claims are also often based on associative changes in signaling pathways without causal validation. Reporting quality is another important limitation, as information on dose rationale, treatment timing, comparator selection, randomization, blinding, and sample size justification is frequently incomplete.

Clinical evidence is even more limited. Available studies are few, mostly single-center, and generally involve small sample sizes. Most focus on short-term biomarkers or general critical care outcomes rather than validated AGI severity measures, gastrointestinal functional recovery, or long-term prognosis. Therefore, current clinical data are insufficient to establish the efficacy of SFI specifically for sepsis-induced AGI.

Further limitations relate to product characterization and safety assessment. As a complex botanical injectable, SFI requires careful consideration of formulation standardization, batch consistency, sterility assurance, endotoxin control, and contamination risk. However, these aspects are insufficiently addressed in much of the current literature, despite their clear relevance to critically ill patients.

Future research should therefore prioritize: (1) standardized and clinically relevant preclinical models; (2) more rigorous mechanistic studies with causal validation; (3) multicenter, adequately powered clinical trials using well-defined sepsis-induced AGI populations and validated gastrointestinal endpoints; and (4) improved characterization of SFI composition, quality control, and safety. Addressing these issues will be essential to determine the true therapeutic value of SFI in sepsis-induced AGI.

## 8 Conclusion

In conclusion, SFI shows potential as an adjunctive therapeutic strategy for sepsis-induced AGI, based on its reported effects on hemodynamics, inflammation, intestinal barrier integrity, and organ protection in preclinical and limited clinical studies. However, the current evidence remains insufficient to support definitive conclusions regarding its efficacy specifically for sepsis-induced AGI. More rigorous mechanistic studies, standardized product characterization, and adequately powered multicenter clinical trials using validated gastrointestinal endpoints are needed before its therapeutic value can be established with confidence.
