# Supplementary figures and images for: Shenfu injection in sepsis-induced acute gastrointestinal injury: a narrative review of mechanisms and current evidence
Source: Front Pharmacol. 2026 May 1;17:1811495. doi: 10.3389/fphar.2026.1811495 (PMC13176289; doi:10.3389/fphar.2026.1811495)

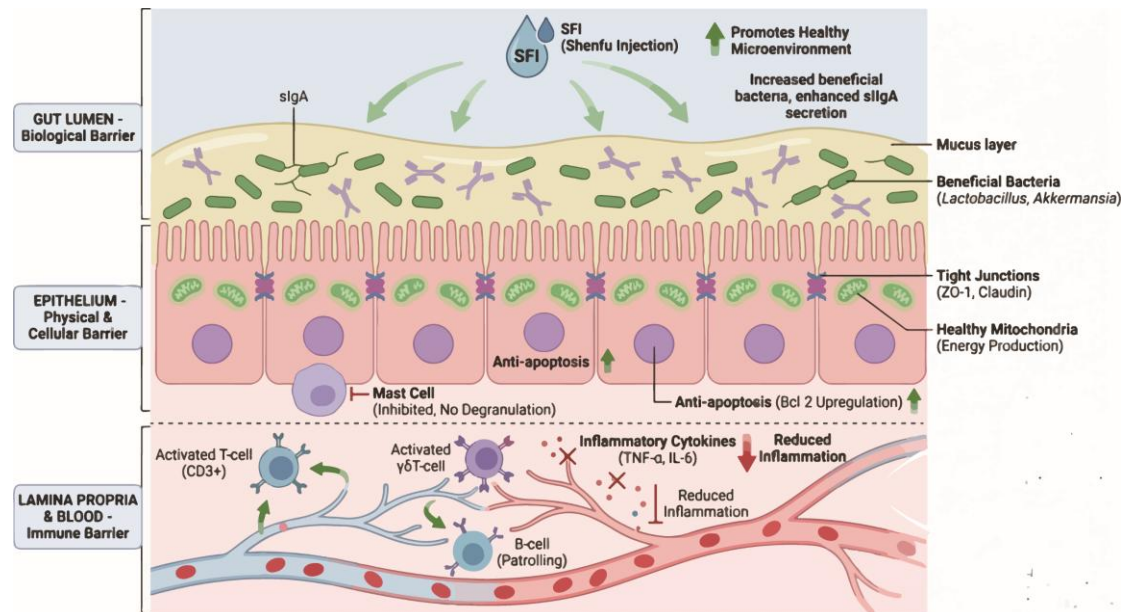

Supplement: Supplementary file 3 [file Supplementaryfile1.zip › Supplementary Material Presentation-figures/Figure3.pdf]

## HEMODYNAMIC IMPROVEMENT & INTESTINAL BARRIER PROTECTION UNDER SFI TREATMENT

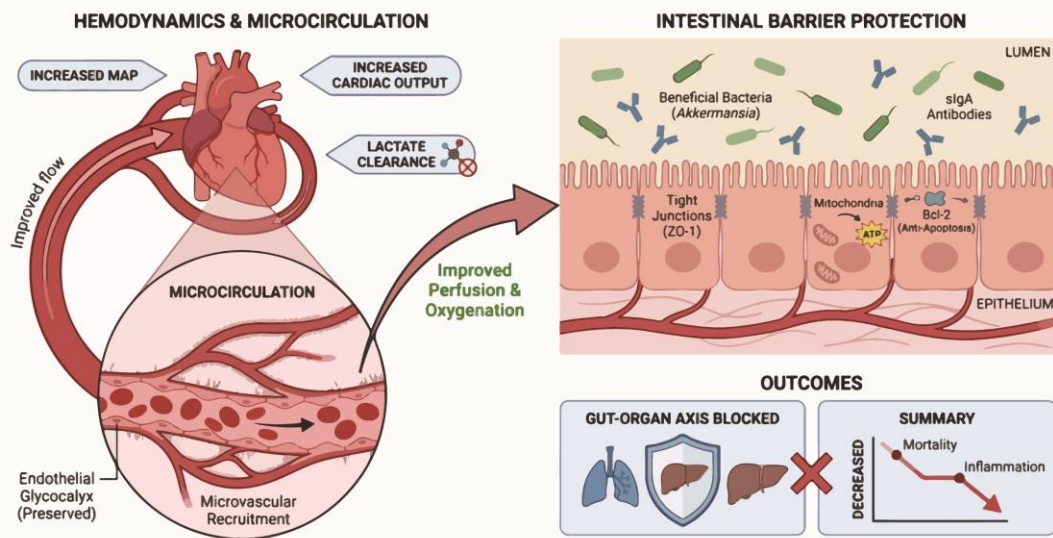

Supplement: Supplementary file 3 [file Supplementaryfile1.zip › Supplementary Material Presentation-figures/Figure2.pdf]

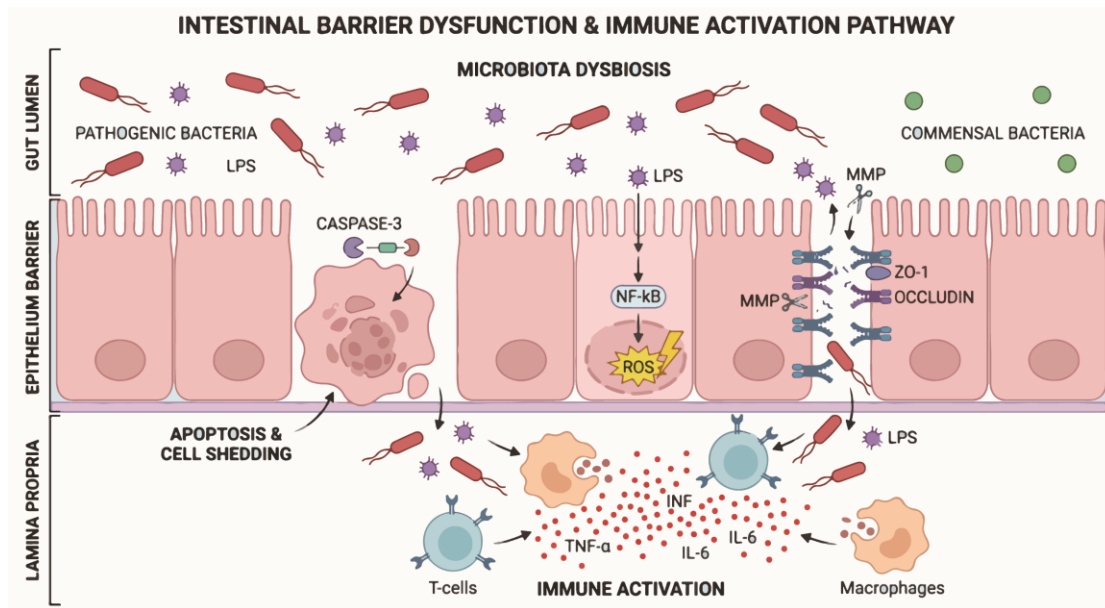

Supplement: Supplementary file 3 [file Supplementaryfile1.zip › Supplementary Material Presentation-figures/Figure1.pdf]
